# Mediating role of coping style on the relationship between job stress and subjective well-being among Korean police officers

**DOI:** 10.1186/s12889-020-08546-3

**Published:** 2020-04-09

**Authors:** Gi Wook Ryu, Yong Sook Yang, Mona Choi

**Affiliations:** Yonsei University College of Nursing, Mo-Im Kim Nursing Research Institute, 50 Yonsei-ro, Seodaemun-gu, Seoul, 03722 Republic of Korea

**Keywords:** Affect, Coping, Job stress, Police officers, Subjective well-being

## Abstract

**Background:**

Police officers have long been known to have one of the most stressful occupations. This study investigates their stress levels, coping styles, and subjective well-being, including affect and life satisfaction. We also explore the interrelationships of these factors to determine how coping style influences a police officer’s subjective well-being.

**Methods:**

We used a convenience sampling method for 112 police officers in a metropolitan area in South Korea. Data were collected using self-administered questionnaires. The questionnaires consisted of the following scales: job stress, coping style, positive/negative affect, and life satisfaction that measured subjective well-being. Descriptive statistics*,* a correlation analysis and Hayes’ PROCESS macro, and bootstrap analysis were performed.

**Results:**

The level of job stress for the participants was moderate, with an average of 43.57 out of 100. The mean scores of positive affect was 17.38 out of 40, 8.50 out of 40 for negative affect, and 20.76 out of 35 for life satisfaction. Job stress and coping were significantly correlated with subjective well-being, and problem-solving style mediated stress, positive affect, and life satisfaction. Lastly, assistance pursuit style mediated stress and positive affect.

**Conclusions:**

Participants’ problem-solving and assistance pursuit coping styles were shown as important mediating factors for stress and subjective well-being, especially positive affect. These findings need to be considered when planning interventions and implementing strategies focusing on the psychosocial health of the improvement of police officers’ well-being.

## Background

Police officers are professionals who respond to emergency situations related to various crimes, such as robbery, sexual assault, and murder [1]. They must face criminals who commit violence, pose a physical threat, and swear at them in the course of their work. Police are also routinely exposed to traumatic events such as traffic accidents, natural disasters, and the witnessing of deaths [2, 3]. Police work has emotional labor characteristics in nature because it requires public contact [4]. In addition to the stressors related to the above circumstances (occupational stressors), the officers also have organizational stressors that include coping with administrative structure, promotion, and working environment [5, 6]. With a hierarchical nature of police organizations, solidarity and socialization are important [3, 7]. Besides, most police officers are engaged in shift work on a 24-h schedule [8]. This can lead to social isolation because of differing patterns of life compared to family and friends [2]. Shift work influences circadian rhythm, affecting hormonal imbalances [8, 9]. In a cohort study of US police officers, a 10-year follow-up of 214 non-depressed police officers found that 10.7% of the subjects became depressed [10]. Taken together, it is clear that police officers have high levels of stress and may face a chronic psychological burden due to work-related demands.

There have been many studies that address the subject of police officers’ stress. Previous reports state that the suicide rate of police officers is higher than that of other careers [2]. The mortality and morbidity rates are also higher when compared with the general population. For example, in the United States, the incidence of cardiovascular disease among police officers was shown to be 31.4%, compared with 18.4% in the general population [11]. The work stress experienced by police officers has a negative impact on their health.

In South Korea, there has recently been an annual increase in the number of emergency calls and reports requiring the attention of police officers [12]. However, there are currently not enough police officers when there is an increasing demand for safety and security, for example, one police officer for every 444 persons in Korea [13]. In comparison, the numbers of police officers are approximately one per 351 persons in the United Stated and one per 347 persons in France [14].

These stressors and occupational environmental factors of police officers facing impact negatively upon an individual’s well-being in terms of their physical and mental health. The notion of “subjective well-being” (SWB) was first presented in Diener’s research. Diener stated that an individual’s happiness is based on subjective judgment, rather than objective criteria [15]. This judgement includes an overall evaluation of their life and emotional experience and emphasizes the subjectivity of happiness. SWB is influenced by multidimensional circumstances such as culture, health, and social relationships [16]. In a working environment, SWB can affect the quantity of sick leave that is taken, the level of productivity, and absenteeism [17]. High SWB is associated with improved working performance, satisfying interpersonal relationships, good health, and community involvement [18]. SWB consists of both cognitive and emotional factors. Cognitive aspects include satisfaction with life and emotional aspects can be evaluated with positive or negative affect [15].

SWB can be affected by stress and coping. Stress is associated with NA, low health status, and interpersonal dissatisfaction [16, 17]. Moreover, stress, coping, and well-being are interacting elements concerning an imbalance between stress and well-being in the cybernetic theory [19]. It is therefore important to achieve balance by using appropriate coping behaviors. According to Lazarus and Folkman’s transactional model of stress, people respond to stress with either problem-focused coping or emotion-focused coping [20]. Coping refers to an individual’s effort to minimize their physical and mental stress. Furthermore, coping appears to be a mechanism for adapting to the effects of stressful events and involves intentional effort. Problem-focused coping includes problem solving and assistance pursuit as active methods to directly approach problems to eliminate stressors. Emotion-focused coping is a passive coping strategy that is a reaction that attempts to control stress-related emotions to maintain an emotional balance. Female crime scene officers typically engage in more emotional support, instrumental support, and positive reframing than male crime scene officers [21]. In a path analysis of police officers’ coping, there was a positive correlation between stress exposure and avoidant coping [1]. In addition, avoidant coping was shown to have an indirect effect on the relationship between stress and well-being. A systematic review of psychological outcomes in police studies showed that avoidant coping strategies negatively affected mental outcomes [3].

**Fig. 1:**
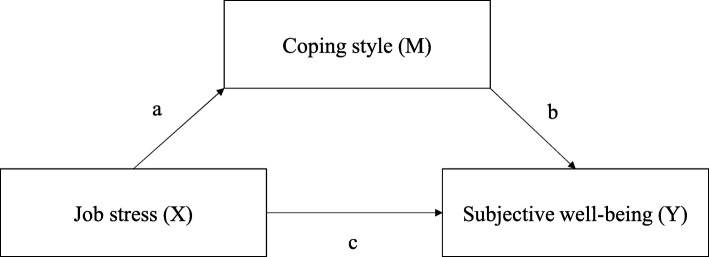
Mediating model of conceptual framework in this study

Although many studies have investigated police officers’ stress, few studies have addressed the relationships among job stress, coping, and SWB. Furthermore, even in the same occupational environment, well-being levels may differ depending on individuals’ responses. However, previous studies have focused on gender differences or did not include emotional variables in SWB. Exploring the roles of coping style as a mediator to the relationship between job stress and SWB is of practical importance, because such knowledge can improve our understanding of police officers’ circumstances, enabling the development of interventions that will be beneficial to their life and work performance. Therefore, this study explores the job stress, coping styles, and SWB of Korean police officers. It examines the relationships between job stress, coping style, and SWB and investigates the mediating role of coping in these relationships. Based on Lazarus and Folkman’s transitional model [20] and Edwards’ cybernetic theory [19], we hypothesized that coping style would act as a mediator in the relationship between job stress and SWB (Fig. 1).

## Methods

### Participants

A cross-sectional study was conducted and aimed at examining the relationship between job stress, coping, and subjective well-being using a cross-sectional survey. The police station where the study was conducted is located in Gyeonggi-do, South Korea, where the population is over 12 million. We used a convenience sampling method to select regions and participants. The participants were police officers between 20 and 60 years old who have worked as police officers for more than 6 months. Individuals previously diagnosed with mental disorders such as depression were excluded. The number of participants was calculated using Optimal Design Plus 3.01 with a median effect size of 0.40, a power of 0.80, and a *p*-value of 0.05. A necessary sample size of 90 individuals were calculated. Data were collected from 112 individuals considering a possible dropout rate of 25%.

### Measures

Participants completed self-reported questionnaires to assess job stress, coping style, affect, and life satisfaction based on the SWB concept. The survey also collected demographic information including age, gender, working duration, position, shift/non-shift, department, and grade.

#### Job stress

The Korean occupational stress scale–short form (KOSS–SF) is a reliable and previously validated scale [22]. This tool was developed during the “National Study for Development and Standardization of Occupational Stress Project” in Korea. The tool was developed with reference to the OSI (occupational stress index), and the NIOSH (National Institute for Occupational Safety and Health) model, and has since been validated by qualitative research, expert opinions, and quantitative research. Additionally, a representative sample of nationwide Korean representatives was selected and standardized measurement tools. The scale consists of 24 items in seven categories: (1) job demand; (2) insufficient job control; (3) interpersonal conflict; (4) job security; (5) organizational system; (6) lack of reward; and (7) occupational climate. Answers are measured with a 4-point Likert scale ranging from “not at all” (1) to “extremely” (4). The method for calculating the score of each category is ((acquired score - number of items)/ (highest possible score - number of items))*100 to convert to a total of 100 points. The total score for job stress is the average of the sum of the scores of each category. A higher score indicates a higher level of job stress experienced by the participant. The Cronbach’ α for this scale was .767.

#### Coping style for stress

The questionnaire for coping style used in this study was translated from English to Korean and has been proven to have good reliability and validity [20, 23]. This tool consists of 39 items used to measure five coping styles: (1) problem-solving style; (2) emotional relieving style; (3) assistance pursuit style; (4) problem avoidance style; and (5) hopeful thinking style. They were measured with a 4-point Likert scale (0–3) ranging from “never” (0) to “always” (3). The score calculation method used was to average the score of each coping style. A higher score for a specific coping style means that the participant often used this style. The Cronbach’ α was .818.

#### Subjective well-being (positive and negative affect)

This study used the positive affect and negative affect schedule (PANAS) [24, 25] that was translated from English to Korean. This tool consists of 20 items that measure two scales: PA and NA. PA refers to a state of high energy, full concentration, and pleasurable engagement, while NA refers to subjective distress and unpleasurable engagement. The items were measured using a 5-point Likert scale ranging from “not at all” (0) to “extremely” (4). The total score was calculated by adding the positive and negative affect scores separately. A higher score means higher positive or negative affect respectively. The Cronbach’ α was .911.

#### Life satisfaction

The satisfaction with life scale (SWLS) [15] used in this study was translated and localized from English to Korean [26]. This scale consists of five items, for which the answers (e.g., “In most ways my life is close to my ideal, and the conditions of my life are excellent”) are ranked from “very low” (1) to 7 “very high” (7). A higher score indicates higher life satisfaction. The Cronbach’s α was .904.

### Data collection

We obtained permission for data collection from the captain of each police station and obtained informed consent from the participants. We visited seven police stations in the study area from August to September, 2018. Data were collected using a self-administered questionnaire. After the participant completed the survey, they submitted it to the research team and received a small gift.

### Statistical analysis

Demographics and work characteristics, job stress, affect, and life satisfaction were analyzed using descriptive statistics. To determine the mediation effect, we identified the relationships between the variables. To do this, the variables of job stress, coping, affect, and life satisfaction were analyzed using a Pearson’s correlation coefficient. We used Hayes’s PROCESS macro and a modal 4 bootstrap method to examine the mediating effect of coping in the relationship between job stress and SWB. Unlike a causal statistical approach, the PROCESS macro approach offers indirect mediating effects and can be applied to a small sample size [27, 28]. In the first step, we defined job stress as a dependent variable (X) and SWB (PA, NA, life satisfaction) as an independent variable (Y) (Fig. 1). In the second step, we tested the mediating effect of X on Y (a × b) and obtained a 95% CI. If the 95% CI did not cross zero, there was a significant mediating effect. For this study, we assumed that SWB includes three elements: PA, NA, and life satisfaction, based on our literature review. We therefore analyzed the mediating effects to determine if there is a correlation among the factors (such as job stress), the sub-categories of coping, and any of the three SWB elements. For data analysis, we used SPSS 23.0 statistics software.

## Results

The participant group’s mean age was 38.9 years old (SD ±11.3) and 95.5% (*n* = 107) were male. Participants with a rank higher than inspector accounted for 40.2% (*n* = 45) and 75% (*n* = 84) of the participants were shift workers. The mean for duration of employment as police officer was 12.2 (± 11.0) years (Table 1).

**Table 1:** General and job-related characteristics (*n* = 112)

Variables	Category	Mean (±SD) or n (%)
Age (years)		38.9 (± 11.3)
Gender	Male	107 (95.5)
	Female	5 (5.5)
Marital status	Single	43 (38.4)
	Married	69 (61.6)
Rank	Police officer	42 (37.5)
	Senior police officer/Assistant Inspector	25 (22.3)
	Inspector or higher	45 (40.2)
Department of work	Public safety division	75 (67.0)
	Detective division	4 (3.6)
	Traffic affairs division, Riot police corps,	27 (24.1)
	Command center, Women & Juvenile affairs division	6 (5.4)
Shift working	Yes	84 (75.0)
	No	28 (25.0)
Working duration (years)		12.2 (± 11.0)

Participants reported moderate levels of job stress, 43.57 (± 8.56) out of 100. Considering the sub-categories (out of 100) of job stress, insufficient job control (55.13 ± 15.83) and organizational system (52.90 ± 15.65) had higher scores higher than the other sub-categories. Interpersonal conflict (33.93 ± 13.24) and job security (36.16 ± 18.97) had the lowest scores. Considering coping strategies (out of 3), problem avoidance style (1.82 ±0.37) and problem-solving style (1.81 ±0.26) had higher means than those of the other categories, such as hopeful thinking (1.80 ±0.37), assistance pursuit (1.73 ±0.29), and emotional relieving (1.36 ±0.26). Considering SWB, the mean scores of PA and NA were 17.38 (±7.27) and 8.50 (±7.79) out of 40 respectively. Life satisfaction was 20.76 (±5.91) out of 35 (Table 2).

**Table 2:** Levels of job stress, coping style, and life satisfaction (*n* = 112)

Variables	Category	Mean ± SD
Job stress	Job demand	42.86 ± 13.92
(Score range: 0–100)	Insufficient job control	55.13 ± 15.83
	Interpersonal conflict	33.93 ± 13.24
	Job security	36.16 ± 18.97
	Organizational system	52.90 ± 15.65
	Lack of reward	43.25 ± 14.41
	Occupational climate	40.77 ± 16.98
	Total mean score	43.57 ± 8.56
Coping style	Problem-solving style	1.81 ± 0.26
(Score range: 0–3)	Emotional relieving style	1.36 ± 0.26
	Assistance pursuit style	1.73 ± 0.29
	Problem avoidance style	1.82 ± 0.37
	Hopeful thinking style	1.80 ± 0.37
Affect	Positive affect	17.38 ± 7.27
(Score range: 0–40)	Negative affect	8.50 ± 7.79
Life satisfaction (Score range: 5–35)		20.76 ± 5.91

Job stress, coping style, PA, and life satisfaction were correlated. Specifically, job stress had a negative correlation with problem-solving style (*r* = −.420, *p* < 0.001), assistance pursuit style (*r* = −.291, *p* = .002), positive affect (*r* = −.237, *p* = .012), and life satisfaction (*r* = −.226, *p* = .017). Positive affect had a significant positive correlation with negative affect (*r* = .337, *p* < 0.001) and life satisfaction (*r* = .436, *p* < 0.001) (Table 3). Based on these results, the paths were drawn to examine the mediating effect (Fig. 1). First, there was a correlation between job stress and problem-solving style (path a). Problem-solving style was correlated with PA and life satisfaction (path b), whereas job stress was correlated with PA and life satisfaction (path c). Second, there was a correlation between job stress and assistance pursuit style (path a) and assistance pursuit style was positively correlated with PA (path b). In addition, job stress was positively correlated with PA (path c).

**Table 3:** Correlations among job stress, coping style, affect (PA, NA), and life satisfaction

*r*									
	1	2	3	4	5	6	7	8	9
1. JS		−.420**	.120	−.291**	−.148	.080	−.237*	.110	−.226**
2. PSS			.025	.387**	.479**	.219*	.370**	−.054	.402**
3. ERS				.003	.275**	.185	.140	.278**	.052
4. APS					.243**	.124	.342**	−.010	.150
5. PAS						.543**	.101	.017	.213*
6. HTS							−.004	.211*	.011
7. PA								.337**	.436**
8. NA									−.096
9. LS									

**p < 0.01, *p < 0.05

*JS* Job stress, *PSS* Problem-solving style, *ERS* Emotion relieving style, *APS* Assistance pursuit style, *PAS* Problem avoidance style, *HTS* Hopeful thinking style, *PA* Positive affect, *NA* Negative affect, *LS* Life satisfaction.

Problem-solving style acted as a mediator between job stress and PA (β = − .117; 95% CI: -.224, -.033) as well as job stress and life satisfaction association (β = − .108; 95% CI: -.193, -.042). This means that problem-solving style had a mediating effect on the relationship between job stress and PA. Assistance pursuit style acted as a mediator between job stress and PA (β = − .074; 95% CI: -.169, -.041). This means that assistance pursuit style had a mediating effect on the relationship between job stress with PA. Assistance pursuit style did not have a significant mediating effect on job stress, but it had a direct and significant effect on life satisfaction (t = − 2.049, *p* = .043) (Table 4).

**Table 4:** Mediating model and the mediation effects of coping style

		PA			NA			LS	
	β(SE)	*t* *(p)*	95% CI	β (SE)	*t*	95% CI	β (SE)	*t* *(p)*	95% CI
PSS									
Indirect X → Y (a × b)	−.117 (.051)	–	−.224, −.033	.004 (.054)	–	−.122, .094	−.108 (.040)	–	−.193, −.042
Direct X → Y (c)	−.085 (.083)	−1.022 (.309)	−.249, .080	.096 (.095)	1.010 (.315)	−.093, .285	−.048 (.067)	−.721 (.473)	−.180, .084
APS									
Indirect X → Y (a × b)	−.074 (.040)	–	−.169. -.014	−.006 (.038)	–	−.098, .061	−.018 (.024)	–	−.076, .018
Direct X → Y (c)	−.128 (.079)	−1.619 (.108)	−.284, .029	.106 (.091)	1.174 (.243)	−.073, .286	−.137 (.067)	−2.049 (.043)	−.270, −.005

*PSS* Problem-solving style, *APS* Assistance pursuit style, *PA* Positive affect, *NA* Negative affect, *LS* Life satisfaction.

## Discussion

This study explored job stress, coping style, and SWB in Korean police officers and analyzed the relationships among these variables. We also examined the mediating role of coping between job stress and SWB. The results show that police officers’ job stress level was moderate. However, in the sub-category of job stress, the scores for insufficient job control and organizational system of the participants were high. This result is consistent with that of previous studies that police officers experience high organizational stress and it is difficult to handle their working situation [29, 30]. It can be said that a police officer’s stress involves operational and organizational aspects. Operational stress is related to activities such as crime prevention, community service, traffic control, patrol activities, and criminal investigation, while organizational stress is related external factors related to the work environment such as inactivity, bureaucratic administration, and relationships with colleagues and superiors [30, 31].

Our results show that problem avoidance as a coping style was used more than other coping styles. Avoidance coping is used when it is difficult to change one’s tasks or occupational environment [26]. This seems to apply to Korean police officers, because they might have some difficulties to control the work environment and hierarchical organizational system. These results are consistent with studies of police officers in Italy and Sweden showing that police officers use avoidance coping in their work [1, 29]. A study of Italian police officers found that the cause of this result was organizational stress, which includes a bravado and masculine culture [29, 32]. In addition, in a systematic literature review, avoidance coping was shown to be related to organizational stress such as can be seen in military service [3, 32]. Police officers’ coping is thought to be due to the overall characteristics of their work worldwide rather than due to national and sociocultural influences.

The results of this study showed that PA was positively correlated with NA. These factors act independently on the nature of affect [24]. In addition, even if NA is high, PA can be enhanced by appropriate coping strategies. The correlation analysis revealed a negative relationship between job stress and SWB, which indicates that high job stress is associated with lower PA and lower life satisfaction. This finding is consistent with previous studies in different populations, similar to those of medical practitioners [33, 34], who have the common occupational characteristics of police personnel because they have to work on critical incidents as first responders, respond to emergency situations to provide safety, engage in dangerous work, and be exposed to high psychological stressor [1, 35, 36].

The results of the mediating model indicate that coping was a strong mediator for job stress and PA as well as job stress and life satisfaction. These results are consistent with those of previous studies that suggest that the factor most influencing police officers’ job stress is adaptive coping strategy [29]. This is similar to a study showing that coping mediates between work demands and psychological tensions [37]. A study on nurses showed that stress and coping strategies play a mediating role in the relationship between work stress and quality of life [36]. Therefore, coping strategy is a significant variable in the relationship between stress and well-being.

The effect of a problem-focused coping method on stress in everyday life was effective in increasing PA [38]. The results also show that the assistance pursuit coping style mediates job stress and PA. The assistance pursuit style as a part of social support can reduce stress and increase life satisfaction in a stressful situation [35]. In the case of police officers, shift work and overtime may negatively influence their family and social life, which can make them vulnerable and in need of appropriate social support. Therefore, strategies to provide social support through family and peer support programs designed for police officers are essential.

PA influences an individual’s adaptive behavior and positive outcome, which may be a solution to a problem with NA [36, 39]. Increasing an individual’s PA is also effective in controlling, eliminating, and preventing NA and important for the physical and mental health and quality of life of individuals [36, 40]. Therefore, to increase police officers’ subjective well-being, it is necessary to consider enhancing PA.

This study contributes to the accumulation of knowledge by measuring the subjective well-being concept of integrating affect and life satisfaction when analyzing the relationship between stress, coping strategies, and life satisfaction. In this study, the results of Lazarus and Folkman’s transitional model [20] are combined with the concept of subjective well-being from Edwards’ cybernetic theory [19] to construct a conceptual framework and analyze the path of concepts. The results demonstrate that coping strategies mediate between job stress and SWB. In particular, positive responses including problem-solving style and assistance-pursuit style mediated between job stress and PA. In previous research that was focused on negative notions of this topic, avoidance coping style and low work control reduced well-being and increased psychological tensions [1, 3, 22]. Conversely, problem-solving style and assistance pursuit coping style were significant variables, indicating the importance of a positive coping style in this study. This finding can be used as a basis for a stress, affect, and life satisfaction-related intervention study.

There are certain limitations to this study. First, there was a significant imbalance of gender due to the higher proportion of male participants, and this fact may preclude its use in any variable analysis of gender differences. Moreover, although the area of this study conducted was a metropolitan area, there is not a high demand for security in those commuter towns. Therefore, there is a lack of information concerning about stress caused by dealing with more serious criminal cases. We did not investigate the potential individual psychological factors that affect coping. Potential factors could, for example, be resilience and internal growth. Future studies should consider collecting data from other regions where the crime rate is high and also investigating other factors related to coping style.

The subjective well-being of police officers is related to mental health [4]. SWB can also affect police organizations and communities, not just the lives of individuals [4]. Low SWB of police officers leads to absenteeism, job-related accidents, and socially negative perceptions of the police. Thus, individual and organizational efforts to improve SWB are important, because environmental stress caused by organizational structures is difficult for an individual to solve. In addition, the job stress and SWB of police officers are thought to be influenced by the characteristics of the police organization rather than by national and sociocultural differences. Therefore, the results of this study can help researchers to understand the mechanism of stress, SWB, and coping strategies of police officers around the world.

## Conclusions

This study provided an initial understanding of the link between coping style and job stress and SWB among Korean police officers. Our results showed that job stress was correlated with coping and SWB. The problem-solving and assistance pursuit coping styles were mediating factors between stress and SWB. This study adds valuable knowledge to the existing knowledge base and it may also be applicable to other areas of occupational stress experienced by police officers.

The findings of this study could provide a foundation for the development of job stress intervention programs to increase coping skills and PA. To enhance SWB of police officers, those intervention programs should be established within police organizations at a regional or national level that assist reducing distress and promoting coping strategies to deal from general issues to critical incidents on their work. Therefore, future research will work toward develop such programs and evaluate their effectiveness to ultimately promote police officers’ physical and mental health.

## Data Availability

The datasets are available from the corresponding author on reasonable request.

## Abbreviations

SWB: Subjective well-being
PA: Positive affect
NA: Negative affect
